# Exploratory rivaroxaban trial for isolated calf deep vein thrombosis with a risk factor of thrombosis extension: an open-label, multicenter, randomized controlled trial

**DOI:** 10.1016/j.rpth.2024.102515

**Published:** 2024-07-14

**Authors:** Yoshito Ogihara, Norikazu Yamada, Daisuke Izumi, Yuichi Sato, Toru Sato, Hitoshi Nakaya, Tatsuya Mori, Satoshi Ota, Midori Makino, Toru Ogura, Satoshi Tamaru, Yuki Nishimura, Takashi Tanigawa, Atsunobu Kasai, Masakatsu Nishikawa, Kaoru Dohi, ISE CALF DVT Study Investigators

**Affiliations:** 1 Department of Cardiology and Nephrology, Mie University Graduate School of Medicine, Tsu, Japan; 2 Department of Cardiology, Kuwana City Medical Center, Kuwana, Japan; 3 Department of Cardiology, Matsusaka Municipal Hospital, Matsusaka, Japan; 4 Department of Cardiology, Matsusaka Chuo General Hospital, Matsusaka, Japan; 5 Department of Cardiology, Ise Red Cross Hospital, Ise, Japan; 6 Department of Cardiology, Suzuka General Hospital, Suzuka, Japan; 7 Clinical Research Support Center, Mie University Hospital, Tsu, Japan

**Keywords:** anticoagulants, vein thrombosis, randomized controlled trial, recurrence, rivaroxaban

## Abstract

**Background:**

Limited evidence exists regarding the incidence of recurrent venous thromboembolism (VTE) in patients diagnosed with isolated distal deep vein thrombosis (DVT) who are at risk of thrombosis extension whether they receive anticoagulation therapy or not.

**Objectives:**

The study aimed to investigate the incidence of recurrent VTE and the impact of rivaroxaban in this patient population.

**Methods:**

This open-label, exploratory, and randomized controlled trial was conducted at 7 centers in Japan between April 2019 and April 2022. Adult patients with isolated distal DVT at risk of thrombosis extension received either rivaroxaban combined with physical therapy or physical therapy alone for 90 days. Whole-leg ultrasound was performed at 14 and 90 days. We assessed a composite outcome of symptomatic or asymptomatic proximal DVT or symptomatic pulmonary embolism as the primary outcome until the end of the treatment period using an intention-to-treat analysis. Major bleeding was evaluated as a key secondary outcome.

**Results:**

Out of 90 enrolled patients, 3 were excluded due to withdrawal of consent; therefore, we analyzed 87 participants. The rivaroxaban group (*n* = 42) reported no primary outcomes (0%; 95% CI, 0.0%-8.4%), whereas the physical therapy group (*n* = 45) had 2 cases of symptomatic proximal DVT (4.4%; 95% CI, 0.5%-15.1%). Major bleeding events occurred in 4 patients in the rivaroxaban group (9.5%; 95% CI, 2.7%-22.6%), whereas no events occurred in the physical therapy group (0%; 95% CI, 0%-7.9%).

**Conclusion:**

Preliminary data suggest that rivaroxaban may reduce the risk of VTE recurrence among this patient subset, albeit with an increased incidence of bleeding events.

## Essentials

• Rivaroxaban’s impact in patients with isolated distal deep vein thrombosis at risk of thrombosis extension has not been well studied.
• An exploratory randomized trial assessed potential effectiveness and safety in such patients.
• Rivaroxaban reduced venous thromboembolism recurrence to 0% over 90 days.
• Rivaroxaban increased bleeding event rates.

## 1 Introduction

Isolated distal deep vein thrombosis (IDDVT) is common, often presenting with a lower recurrence rate of venous thromboembolism (VTE) than of proximal deep vein thrombosis (DVT) especially in asymptomatic cases [1,2]. However, symptomatic IDDVT patients with risk factors for thrombosis extension, such as active cancer or severe symptoms, experience a higher frequency of recurrent VTE [[3], [4], [5], [6]]. Thus, international guidelines suggest anticoagulation therapy for at-risk IDDVT patients [[7], [8], [9]]. Despite this, evidence from randomized clinical trials (RCTs) is limited, and the necessity of anticoagulation therapy for asymptomatic but at-risk IDDVT patients remains unclear, making the optimal management controversial.

Our exploratory study aimed to investigate the incidence of recurrent VTE in at-risk IDDVT patients irrespective of symptoms. We also evaluated the potential effectiveness and safety of rivaroxaban anticoagulation therapy in this subgroup, contributing to identifying suitable candidates for anticoagulation therapy among IDDVT patients and suggesting future research directions for refining treatment guidelines.

## 2 Methods

### 2.1 Study design and oversight

The ISE CALF DVT study was an open-label, adjudicator-blinded study across 7 institutions in 5 cities within Mie Prefecture, Japan (Supplementary Appendix 1 and 2). Participants were randomized to receive either rivaroxaban plus physical therapy or physical therapy alone.

The trial protocol was approved by the Certified Review Board of Mie University (approval number S2018-001) and the corresponding boards at the participating centers and adhered to the Declaration of Helsinki and the Japanese Clinical Trials Act. Written informed consent was obtained from all participants. The trial was registered in the Japan Registry of Clinical Trials (ID: jRCTs041190009; https://jrct.niph.go.jp/latest-detail/jRCTs041190009). An independent committee, blinded to the treatment assignments, adjudicated all prespecified events. This study follows the Consolidated Standards of Reporting Trials guideline (Supplementary Table S1).

### 2.2 Patients

Detailed definition of patient characteristics and inclusion/exclusion criteria are available in Supplementary Appendix S4.

Adults (aged ≥20 years) newly diagnosed with IDDVT through whole-leg ultrasound (US) within 3 days prior to enrollment were eligible. Eligibility required at least 1 risk factor for thrombus extension as referenced in the American College of Chest Physicians’ guidelines [7], including symptoms such as swelling and pain; a large thrombus or a thrombus near the popliteal vein; active cancer; previous proximal DVT or pulmonary embolism (PE); or ≥72 hours of bed rest during hospitalization. As a result, the study included asymptomatic IDDVT patients identified through screening who presented these risk factors. Whole-leg US, assessing all lower extremity veins, was performed by experienced sonographers following standard practice. IDDVT diagnosis relied on detecting incompressibility in the distal veins (peroneal, tibial, soleus, or gastrocnemius veins) under compression.

Exclusion criteria included patients with contraindications to rivaroxaban such as a significant liver or kidney disease or pregnancy. Additional exclusions were acute PE or proximal DVT, need for anticoagulants for other conditions, severe health issues with life expectancy under 3 months, or inability to use graduated compression stockings (GCSs) or elastic bandages.

### 2.3 Randomization and study intervention

The patient registration, randomization, and study procedures are detailed in Supplementary Appendix 5.

Participants were enrolled by investigators through a web-based system administered by the data center and randomly assigned in a 1:1 ratio to receive either rivaroxaban plus physical therapy (rivaroxaban group) or physical therapy alone (physical therapy group). Randomization used a minimization method with adjustment factors of active cancer, planned surgery, and institution.

The rivaroxaban group was planned to receive 15 mg orally twice daily for the first 21 days, followed by 15 mg once daily up to day 90 (±14 days), as per the Japanese product label [10]. However, the protocol allowed for starting with 15 mg once daily from the outset at the investigator’s discretion due to concerns such as high bleeding risk. All participants received physical therapy, including wearing knee-length GCSs (ankle pressure of 15-30 mmHg), regular walking, and ankle exercises.

Rivaroxaban administration was suspended due to medical necessity, such as invasive treatment or adverse events, with appropriate measures taken based on clinical practice. After resolving events, the investigator determined whether to resume or discontinue rivaroxaban. Participants were considered adherent if their rivaroxaban administration rate was at least 80%.

### 2.4 Surveillance and follow-up

This study comprised an intervention period from day 1 to day 90 and a follow-up period up to day 365. During the intervention period, whole-leg US was conducted on day 14 (±3 days) and day 90 (±14 days) to monitor thrombus status. Investigators verbally confirmed treatment adherence at each follow-up visit. Suspected recurrent VTE events, including PE or proximal DVT (encompassing cases where IDDVT extended into proximal veins), were confirmed with additional imaging techniques, such as computed tomography, pulmonary arteriography, lung scintigraphy, or whole-leg US.

### 2.5 Endpoints

The primary outcome comprised a composite of symptomatic or asymptomatic proximal DVT or symptomatic PE (including fatal cases), assessed during the visit on day 90 (±14 days). Secondary outcomes included symptomatic PE, symptomatic or asymptomatic proximal DVT, recurrent IDDVT, major bleeding, clinically relevant bleeding (encompassing major and clinically relevant nonmajor bleeding), and all-cause mortality. These outcomes adhered to prespecified criteria (Supplementary Appendix S6). Major bleeding was defined according to the International Society on Thrombosis and Haemostasis standards [11]. Clinically relevant nonmajor bleeding was defined as events that did not qualify as major bleeding but included those requiring medical intervention, unscheduled visits, telephone inquiries, or the discontinuation of rivaroxaban. This category also included events that caused symptoms such as pain and interfered with activities of daily living [12]. In the safety analysis, adverse events, including bleeding and death, were monitored through 2 days after the last intervention. Two patients in the physical therapy group missed their day 90 visit; therefore, their data for this time point were imputed using information from their next available visit, during which no primary events or bleeding events were recorded.

### 2.6 Statistical analysis

Due to the unique characteristics of our study population—predominantly asymptomatic yet at risk for VTE extension—no statistical tests were planned for group comparisons in this exploratory study. The target of enrolling 150 patients over a 1.5-year period was determined based on historical data from the participating facilities, reflecting the study’s practical feasibility.

Categorical variables were presented as numbers and percentages; continuous variables were presented as mean and SD or median and IQR. The incidence of primary and secondary outcomes was calculated as the proportion of patients experiencing at least 1 event, relative to the total number of patients in the intention-to-treat analysis set. The 2-sided 95% CI for the rate of occurrence was calculated using the Clopper–Pearson method. All statistical analyses were performed using SAS software version 9.4 (SAS Institute Inc).

## 3 Results and Discussion

Between April 10, 2019, and April 30, 2022, 90 patients were enrolled. Despite an extended enrollment period, the number of participants remained below expectations, largely due to the COVID-19 pandemic’s impact. Due to financial constraints, extending the enrollment period was not feasible. Three patients withdrew after randomization; therefore, 87 patients remained in the intention-to-treat set. The patients were divided into 42 in the rivaroxaban group and 45 in the physical therapy group (Figure). The study population predominantly consisted of patients with active cancer (67.8%), advanced age (≥75 years; 58.6%), inpatient status (47.1%), anemia (57.5%), and positive D-dimer results (94.3%), with infrequent IDDVT-related symptoms (10.3%; Table 1). Primary reasons for performing USs were elevated VTE risk (81.6%) and perioperative screening (37.9%; Supplementary Table S2).

**Figure:**
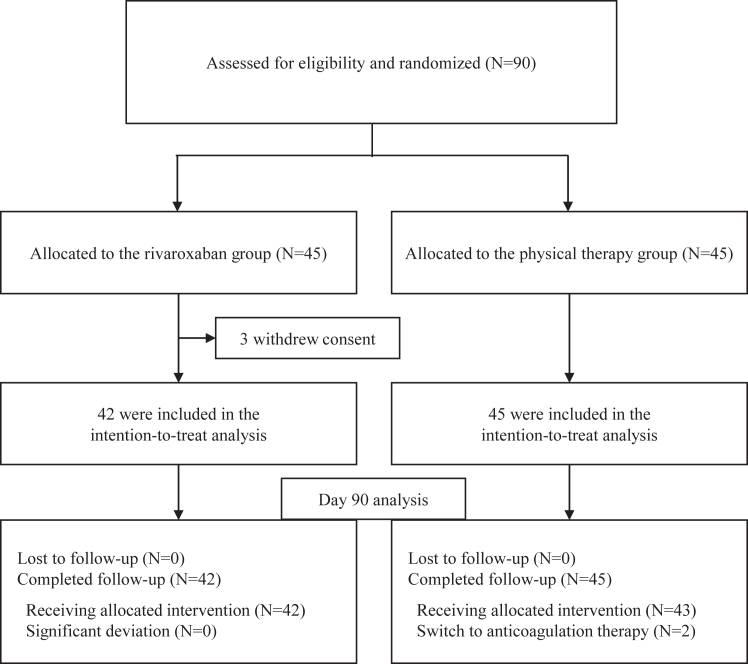
Study flowchart. Participant progression through the study. In the rivaroxaban group, participants adhered to the prescribed treatment protocol without significant deviations. On the other hand, in the physical therapy group, 2 participants required a switch to anticoagulation therapy due to changes in their medical conditions.

**Table 1:** Patient characteristics at baseline and treatment.

Characteristic	Rivaroxaban (*n* = 42)	Physical therapy (*n* = 45)
Age (y)	76 (72-80)	75 (70-79)
Age ≥75 y	28 (66.7)	23 (51.1)
Women	31 (73.8)	24 (53.3)
Body weight (kg)	53.5 (46.0-60.0)	57.9 (51.3-69.0)
Body mass index (kg/m^2^)	22.8 ± 4.3	23.7 ± 3.2
Location of calf DVT		
Soleus vein	39 (92.9)	41 (91.1)
Peroneal vein	7 (16.7)	11 (24.4)
Posterior tibial vein	0 (0)	4 (8.9)
Gastrocnemius vein	2 (4.8)	1 (2.2)
Risk factors for IDDVT extension		
IDDVT-related symptoms	6 (14.3)	3 (6.7)
Pain	3 (7.1)	1 (2.2)
Swelling	5 (11.9)	3 (6.7)
Large thrombosis	25 (59.5)	31 (68.9)
Thrombosis close to the proximal veins	2 (4.8)	1 (2.2)
Active cancer	29 (69.0)	30 (66.7)
Under chemotherapy	15 (35.7)	20 (44.4)
History of VTE	1 (2.4)	2 (4.4)
Immobility	3 (7.1)	2 (4.4)
Treatment for DVT		
Initial treatment of rivaroxaban		
15 mg twice daily	0 (0)	-
15 mg once daily	42 (100)	-
Physical therapy		
Use of GCS 3 or more days per week	43 (95.6)	38 (90.5)
The other underlying conditions		
Inpatient	28 (66.7)	13 (28.9)
Scheduled for surgery	11 (26.2)	12 (26.7)
Paralysis	3 (7.1)	5 (11.1)
Trauma and/or fracture	2 (4.8)	2 (4.4)
Recent surgery	8 (19.0)	7 (16.3)
Prior stroke	4 (9.5)	4 (8.9)
Acute infection	5 (11.9)	3 (6.7)
Heart failure	2 (4.8)	1 (2.2)
Chronic lung disease^a^	2 (4.8)	4 (8.9)
Hypertension	20 (47.6)	26 (57.8)
Diabetes	4 (9.5)	8 (17.8)
Dyslipidemia	14 (33.3)	12 (26.7)
Chronic inflammatory disease	3 (7.1)	3 (6.7)
Antiphospholipid syndrome	0 (0)	1 (2.2)
History of major bleeding^b^	2 (4.8)	3 (6.7)
Laboratory tests		
Anemia^c^ (<13 g/dL for men and <12 g/dL for women)	22 (52.4)	28 (62.2)
Thrombocytopenia^d^ (platelet count <100,000/μL)	1 (2.4)	0 (0)
CrCl levels^e^ (mL/min)	62.4 (51.9-79.2)	61.7 (46.8-81.4)
D-dimer (μg/mL)	3.3 (1.7-7.9)	3.5 (1.8-7.0)
Positive D-dimer result (≥1.0 μg/mL)	39 (92.9)	43 (95.6)
Concomitant medications		
Antiplatelet agents	4 (9.5)	6 (13.3)
Nonsteroidal anti-inflammatory drugs	5 (11.9)	10 (22.2)
Proton pump inhibitors/H2 blockers	18 (42.9)	14 (31.1)
Statins	7 (16.7)	8 (17.8)

Categorical variables are presented as numbers and percentages, and continuous variables are presented as mean and SD or median and IQR based on their distributions.

CrCl, creatinine clearance; DVT, deep vein thrombosis; GCS, graduated compression stocking; H2 blockers, histamine-2 receptor blockers; IDDVT, isolated distal deep vein thrombosis; VTE, venous thromboembolism.

a Chronic lung disease was defined as persistent lung disorders such as asthma, chronic obstructive pulmonary disease, and restrictive lung diseases.

b History of major bleeding was diagnosed if the patient had a history of major bleeding according to International Society on Thrombosis and Haemostasis criteria.

c Anemia was defined as hemoglobin level <13 g/dL for men and <12 g/dL for women.

d Thrombocytopenia was defined as platelet count <100 × 10^9^/L.

e Creatinine clearance level was calculated using the Cockroft–Gault formula.

None of the participants received the initial 15 mg twice daily dosing. All started 15 mg once daily, based on high bleeding risk or when thrombosis extension risk was not considered particularly high (Supplementary Table S3). Two patients in the physical therapy group required anticoagulation: one for a left subclavian venous thrombosis after randomization and another due to newly detected paroxysmal atrial fibrillation (Figure). Over 90% of participants in both groups adhered to wearing GCS for 3 or more days weekly (Table 1). Follow-up ultrasonography at day 14 did not alter the initial treatment plans, and protocol adherence was maintained despite temporary suspensions of rivaroxaban due to bleeding events (Supplementary Table S4).

No primary outcomes were reported in the rivaroxaban group (0%; 95% CI, 0.0%-8.4%). In the physical therapy group, 2 symptomatic proximal DVTs occurred (4.4%; 95% CI, 0.5%-15.1%): one ipsilateral and the other contralateral to the initial IDDVT sites. Active cancer was a risk factor in both primary outcome cases. Major bleeding occurred in 4 patients in the rivaroxaban group (9.5%; 95% CI, 2.7%-22.6%); however, there were no events in the physical therapy group (0%; 95% CI, 0%-7.9%). No deaths were observed between day 0 and day 90 in either group (Tables 2 and 3).

**Table 2:** Efficacy and safety clinical outcomes at day 90 (±14 days).

Outcome	Rivaroxaban (*n* = 42)		Physical therapy (*n* = 45)		Absolute risk reduction (%; 95% CI)^b^
	*n* (%)	95% CI^a^	*n* (%)	95% CI^a^	
Primary outcome					
Composite outcome of symptomatic or asymptomatic proximal DVT or symptomatic PE	0 (0)	0 to 8.4	2 (4.4)	0.5 to 15.1	4.4 (−4.6 to 15.1)
Secondary outcomes					
Thrombotic events					
Symptomatic PE and proximal DVT	0 (0)	0 to 8.4	2 (4.4)	0.5 to 15.1	4.4 (−4.6 to 15.1)
Symptomatic PE	0 (0)	0 to 8.4	0 (0)	0 to 7.9	0
Symptomatic proximal DVT	0 (0)	0 to 8.4	2 (4.4)	0.5 to 15.1	4.4 (−4.6 to 15.1)
Asymptomatic proximal DVT	0 (0)	0 to 8.4	0 (0)	0 to 7.9	0
Symptomatic or asymptomatic IDDVT	6 (14.3)	5.4 to 28.5	11 (24.4)	12.9 to 39.5	10.2 (−7.5 to 27.6)
Bleeding events					
Major bleeding	4 (9.5)	2.7 to 22.6	0 (0)	0 to 7.9	−9.5 (−22.8 to −0.7)
Clinically relevant bleeding	7 (16.7)	7.0 to 31.4	1 (2.2)	0.1 to 11.8	−14.4 (−29.8 to −2.0)
All-cause death	0 (0)	0 to 8.4	0 (0)	0 to 7.9	0

Categorical variables are presented as numbers and percentages. Regarding the primary and secondary endpoints, the rate of the occurrence of events is reported as the number of patients developing at least 1 event divided by the total number of patients in the intention-to-treat analysis set. Absolute risk reduction is calculated as the event rate in the physical therapy group minus the event rate in the rivaroxaban group. Clinically relevant bleeding comprises major bleeding and clinically relevant nonmajor bleeding.

DVT, deep vein thrombosis; IDDVT, isolated distal deep vein thrombosis; PE, pulmonary embolism.

a The 95% CI of the rate of occurrence was calculated using the Clopper–Pearson method.

b The 95% CI for the risk difference is based on the score statistic.

**Table 3:** Summary of primary outcomes or major bleeding events.

Events	Age (y), sex	Treatment group	Risk factors for events	Onset	Event type
Primary outcome	75, female	Physical	Large thrombus	Day 98	Symptomatic proximal DVT
			Active cancer^a^		
	73, male	Physical	Active cancer	Day 90	Symptomatic proximal DVT
Major bleeding event	70, female	Rivaroxaban	Active cancer	Day 73	Cerebral hemorrhage associated with brain metastasis of melanoma^b^
			Brain metastasis		
			Anemia		
	72, male	Rivaroxaban	Active cancer^c^	Day 11	Cerebral hemorrhage associated with brain metastasis of lymphoma^b^
			Brain metastasis		
			Anemia		
	75, female	Rivaroxaban	Active cancer^c^	Day 76	Gastrointestinal bleeding associated with gastric lesion of lymphoma
			Advanced age		
			Prior major bleeding anemia		
			Thrombocytopenia		
	72, female	Rivaroxaban	Anemia	Day 69	Gastrointestinal bleeding of unknown origin

DVT, deep vein thrombosis; Physical, physical therapy group; Rivaroxaban, rivaroxaban group.

a A 75-year-old female, enrolled 8 months after surgery for cholangiocellular carcinoma, initially presented with a large thrombus in her left soleus vein (measuring 6 cm). However, during the intervention period, she experienced a complication of recurrent cancer followed by proximal DVT.

b Two patients experienced asymptomatic cerebral hemorrhage associated with brain metastases, detected incidentally.

c Cancer in this study comprised all malignant tumors, including hematologic malignancy.

We prospectively collected clinical data during the follow-up period up to day 365. Detailed results from this extended follow-up period are provided in Supplementary Table S5.

Main findings in this study are as follows: the incidence of the composite outcome (proximal DVT or symptomatic PE) in IDDVT patients at risk of thrombus extension upon comparing the outcomes of rivaroxaban treatment with those of physical therapy alone for 3 months was 0% vs 4.4%, respectively, but with a high incidence of bleeding events in the rivaroxaban group.

Prior studies involving symptomatic IDDVT patients identified VTE recurrence rates of 3.8% to 11.4% without anticoagulation [5,13,14], contrasting with a 1.6% recurrence rate in asymptomatic IDDVT patients not receiving anticoagulation [15]. In our study, the 4.4% incidence of primary outcomes in the physical therapy group appears higher than the 1.6% in the previous study of asymptomatic patients. This discrepancy can be attributed to the fact that, although approximately 90% of our study population was asymptomatic, all participants had at least 1 risk factor for thrombus extension. Furthermore, the 2 patients who experienced primary outcomes, despite lacking IDDVT-related symptoms, had active cancer, indicating an elevated risk of recurrent VTE. Based on these findings and using the 0.7% incidence reported in a previous study [16], we projected that a sample size of 302 participants would be necessary for a definitive trial to accurately assess rivaroxaban’s efficacy, assuming a 2-sided alpha of 0.05 and a statistical power of 80%.

Previous studies reported that active cancer significantly increases the risk of anticoagulation-related bleeding [[17], [18], [19], [20]]. Recent RCTs and meta-analyses have provided comprehensive insights into the bleeding risks associated with direct oral anticoagulants in VTE patients with active cancer [[21], [22], [23], [24], [25]]. Despite the high bleeding risk status, direct oral anticoagulants have demonstrated safety comparable with that of standard therapy with low-molecular-weight heparin. Two RCTs involving rivaroxaban reported a 3 to 6-month cumulative major bleeding incidence of 1.4% to 6% [23]. However, the rate of major bleeding in our rivaroxaban cohort was higher (9.5%), likely due to the high proportion of participants with active cancer and other risk factors such as advanced age and anemia. Specifically, 3 of the 4 patients with major bleeding had active cancer and additional known risk factors, including advanced age, brain metastasis, anemia, thrombocytopenia, and prior major bleeding [18,19,[26], [27], [28], [29]]. Given these findings, it may be challenging to further stratify patients with lower bleeding risk within our study population. Considering that the rivaroxaban group had no primary endpoint events despite using 15 mg once daily, exploring a lower dose such as 10 mg daily could be a reasonable approach for future studies. Additionally, newer anticoagulants like factor XIa inhibitors may offer a reduced risk of bleeding [30]. Therefore, future studies could consider evaluating these alternatives to optimize the risk-to-benefit ratio for patients with IDDVT.

The study had several limitations, including its exploratory design, small sample size, and the specific characteristics of our study population. Most participants were asymptomatic, with a high proportion having active cancer, advanced age, inpatient status, anemia, or recent or upcoming surgery. Furthermore, the guidelines do not recommend routine DVT screening for asymptomatic patients, even with thrombosis extension risk [[7], [8], [9]], which were included in our study. This may limit the applicability of our findings to the general population. Therefore, the results should be regarded as preliminary and interpreted with caution.

In conclusion, this exploratory study provides insight into VTE recurrence in IDDVT patients at risk of thrombosis extension treated with rivaroxaban vs physical therapy alone. Our preliminary data indicate that rivaroxaban may reduce VTE recurrence but is associated with a higher incidence of bleeding events. These findings highlight the need for further studies to assess both the efficacy and safety of rivaroxaban in managing IDDVT, ensuring a balanced approach to its clinical application.

## Appendices

Department of Cardiology and Nephrology, Mie University Graduate School of Medicine, Tsu, Japan (Yoshito Ogihara, Hitoshi Nakaya, Toru Sato, Kaoru Dohi). Department of Cardiovascular Medicine, Kuwana City Medical Center, Kuwana, Japan (Norikazu Yamada). Department of Cardiology, Suzuka General Hospital, Suzuka, Japan (Satoshi Ota, Midori Makino). Department of Cardiology, Matsusaka Chuo General Hospital, Matsusaka, Japan (Takashi Tanigawa, Yuichi Sato). Department of Cardiology, Matsusaka Municipal Hospital, Matsusaka, Japan (Daisuke Izumi). Department of Cardiology, Saiseikai Matsusaka General Hospital, Matsusaka, Japan (Hitoshi Kakimoto, Shinya Kato). Department of Cardiology, Japanese Red Cross Ise Hospital, Ise, Japan (Atsunobu Kasai, Tatsuya Mori).
