# Supplementary material for: Exploratory rivaroxaban trial for isolated calf deep vein thrombosis with a risk factor of thrombosis extension: an open-label, multicenter, randomized controlled trial
Source: Res Pract Thromb Haemost. 2024 Jul 14;8(5):102515. doi: 10.1016/j.rpth.2024.102515 (PMC11347048; doi:10.1016/j.rpth.2024.102515)
Supplement: Supplemental File [file mmc1.docx]

**Supplementary File**

**Table of Contents**

[**Supplementary Appendix** 3](#_Toc169992428)

[**Supplementary Appendix1: Study organization** 3](#_Toc169992429)

[**Supplementary Appendix 2: Participating centers and investigators** 5](#_Toc169992430)

[**Supplementary Appendix 3: Abbreviations** 6](#_Toc169992431)

[**Supplementary Appendix 4: Detailed definitions of patient’s characteristics and inclusion/exclusion criteria** 7](#_Toc169992432)

[**Definitions** 7](#_Toc169992433)

[**Inclusion criteria** 7](#_Toc169992434)

[**Exclusion criteria** 8](#_Toc169992435)

[**Supplementary Appendix 5: Study procedure** 10](#_Toc169992436)

[**Patient registration procedure** 10](#_Toc169992437)

[**Randomization procedure.** 10](#_Toc169992438)

[**Procedure** 11](#_Toc169992439)

[**Supplementary Appendix 6: Endpoints and Definition of the Endpoints** 13](#_Toc169992440)

[**Primary endpoint** 13](#_Toc169992441)

[**Secondary endpoint** 13](#_Toc169992442)

[**Definitions for endpoint** 13](#_Toc169992443)

[**Supplementary Tables** 16](#_Toc169992444)

[**Table S1: Consort 2010 checklist compliance for our randomized trial** 16](#_Toc169992445)

[**Table S2. Reasons for performing ultrasound** 19](#_Toc169992446)

[**Table S3. Reasons for selecting the initial lower dosing of rivaroxaban** 20](#_Toc169992447)

[**Table S4. Efficacy and safety clinical outcomes at Day 14 (± 3 days)** 21](#_Toc169992448)

[**Table S5. Efficacy and safety clinical outcomes at Day 365 (± 28 days)** 22](#_Toc169992449)

# **Supplementary Appendix**

## **Supplementary Appendix1: Study organization**

**Chief Investigator.**

Yoshito OGIHARA, Department of Cardiology and Nephrology, Mie University Graduate School of Medicine.

**Lead statistician.**

Toru OGURA, Clinical Research Support Center, Mie University Hospital

**Patient registration center, data center, and data management officers**

Yuki NISHIMURA, Clinical Research Support Center, Mie University Hospital; Yasuto IMAI, Clinical Research Support Center, Mie University Hospital: Shoichi MAGAWA, Clinical Research Support Center, Mie University Hospital

**Steering Committee.**

Norikazu YAMADA, Department of Cardiovascular Medicine, Kuwana City Medical Center.

Masakatsu NISHIKAWA, Mie Division, General incorporated foundation KKC (Kinki Kenko Kanri Center).

Satoshi OTA, Department of Cardiology, Suzuka General Hospital.

**Monitoring officer.**

Koji KAWAGUCHI, Clinical Research Support Center, Mie University Hospital

**Audit officer.**

Yoko UHIRA, Specified non-profit corporation, Mie Clinical Trials Support Network

**Clinical Events Committee.**

Mashio NAKAMURA, Hidamarinooka Nakamura Medical Clinic; Hideo WADA, Department of General and Laboratory Medicine, Mie Prefectural General Medical Center.

**Data and Safety Monitoring Committee.**

Satoshi TAMARU, Clinical Research Support Center, Mie University Hospital; Toshihiro KANEKO, Department of Patient Safety and Infection Control, Mie University Graduate School of Medicine; Kana MIYAZAKI, Department of Hematology and Oncology, Mie University Graduate School of Medicine.

## **Supplementary Appendix 2: Participating centers and investigators**

- Department of Cardiology and Nephrology, Mie University Graduate School of Medicine, Tsu, Japan: Yoshito OGIHARA, Hitoshi NAKAYA, Toru SATO, Kaoru DOHI
- Department of Cardiovascular Medicine, Kuwana City Medical Center, Kuwana, Japan: Norikazu YAMADA
- Department of Cardiology, Suzuka General Hospital, Suzuka, Japan: Satoshi OTA, Midori MAKINO
- Department of Cardiology, Matsusaka Chuo General Hospital, Matsusaka, Japan: Takashi TANIGAWA, Yuichi SATO
- Department of Cardiology, Matsusaka Municipal Hospital, Matsusaka, Japan: Daisuke IZUMI
- Department of Cardiology, Saiseikai Matsusaka General Hospital, Matsusaka, Japan: Hitoshi KAKIMOTO, Shinya KATO
- Department of Cardiology, Japanese Red Cross Ise Hospital, Ise, Japan: Atsunobu KASAI, Tatsuya MORI

## **Supplementary Appendix 3: Abbreviations**

CI confidence interval

DOAC direct oral anticoagulant

DVT deep vein thrombosis

GCS graduated compression stocking

IDDVT isolated distal deep vein thrombosis

ITT intention-to-treat

PE pulmonary embolism

RCT randomized clinical trials

US ultrasound

VTE venous thromboembolism

## **Supplementary Appendix 4: Detailed definitions of patient’s characteristics and inclusion/exclusion criteria**

### **Definitions**

Isolated distal DVT is defined as the presence of an incompressible venous segment detected by whole-leg ultrasonography (US) in deep calf veins: posterior tibial, peroneal, anterior tibial, gastrocnemius or soleus veins. Proximal DVT is defined as a thrombus in or extending to the popliteal, femoral, iliac veins, or inferior vena cava. Cancer in the present study comprises all malignant tumor including hematologic malignancy. Additionally, active cancer meets one of the following criteria: cancer diagnosed within six months before enrolment; cancer received any treatment within the previous six months or receiving; recurrent or metastatic cancer; or cancer without complete remission (if hematological malignancy)

### **Inclusion criteria**

Patients with isolated distal DVT newly diagnosed by whole-leg US who fulfill the following criteria:

(1) Males and females aged 20 years or older

(2) Patients who have provided written consent

If the patient has difficulty in registering although he/she is willing to participate in the study due to conditions such as post-infarction hemiplegia, it is permitted for a witness to sign the consent form as a surrogate after confirming the patient’s will. The witness must be the patient’s spouse/partner or a relative within the third degree of relationship.

(3) Patients who have at least one of the following conditions listed as risk factors of progression of isolated distal DVT by the 9th edition of the ACCP guidelines:

a.Having swelling/pain

b. Having a large volume of thrombus or a thrombus adjacent to the popliteal vein (e.g., thrombus extending over 5 cm or more, thrombi present in multiple veins, or thrombus in a vein dilated to a diameter of 7 mm or more)

c. Having active malignant disease

d. Having a history of proximal DVT or PE

e. Being hospitalized　*only when bed rest for 72 hours or longer is required

### **Exclusion criteria**

(1) Patients previously enrolled to this study

(2) Patients in whom anticoagulant therapy has been initiated or is expected to be initiated

(3) Patients with diseases other than DVT that are indications for anticoagulants

(4) Patients with contraindications for graduated compression stockings or elastic bandage

(5) Patients with a history of hypersensitivity to rivaroxaban

(6) Patients with contraindications for rivaroxaban listed below:

a. Active bleeding (clinically relevant bleeding such as intracranial and gastrointestinal bleeding)

b. Child-Pugh B or C liver failure

c. Severe kidney disorder (CrCl <30 mL/min)

d. Pregnant, possibly pregnant, and breast-feeding women

e. Patients during treatment with an HIV protease inhibitor*

f. Patients during treatment with a preparation containing cobicistat*

g. Patients during treatment with an oral or injection preparation of an azole antibiotic*

h. Patients with acute bacterial endocarditis

*These drugs may interact with rivaroxaban by suppressing CYP3A4 and pGP.

(7) Patients who have recently undergone surgery for the central nervous system or have a recent episode of cerebral hemorrhage

(8) Patients with symptomatic acute PE or symptomatic or asymptomatic acute proximal DVT

(9) Patients with serious complications considered to have a life expectancy of less than 3 months

(10) Patients with poorly controlled hypertension (>180 mmHg systolic or >110 mmHg diastolic)

(11) Patients who have participated in a clinical trial of another drug or medical device within 30 days before allocation. Participation in observational studies is permitted.

(12) Patients judged by the investigator to be inappropriate as participants of this study

## **Supplementary Appendix 5: Study procedure**

### **Patient registration procedure**

(1) The investigator explains the study orally and in writing using explanatory documents to patients confirmed to have isolated distal DVT by whole-leg US and obtains written consent. However, the witness is permitted to sign the consent form for the participant after confirming oral consent of the participant if the patient is willing to participate in the study but is difficult to sign the document due to hemiplegia after cerebral infarction, etc. In this event, the witness must write the reason for the inability of the participant to sign the document in person and the relationship between the participant and the witness in the margin, etc., of the consent document.

(2) The patients are confirmed to meet the inclusion criteria and none of the exclusion criteria.

(3) Patients confirmed to be eligible are registered using the clinical research data management system run by aa independent data center, the Clinical Research Support Center, Mie University Hospital, within 72 hours after the diagnosis.

(4) Registration numbers are issued by the data management system. Registration is completed by the issuance of a registration number. Registration at the website ia possible 24 hours a day, 365 days a year.

(5) If retraction of consent, discontinuation, or dropout has occurred, it must be promptly reported to the study secretariat and data center.

### **Randomization procedure.**

The participants are randomly assigned to two groups, Rivaroxaban administration + physical therapy group (rivaroxaban group) or Physical therapy group, by the data center at registration. Randomization is made by the minimization method to balance for the following factors: (1) the presence or absence of active malignant disease; (2) the presence or absence of scheduled surgery; and (3) study institution as adjustment factors to avoid large bias.

### **Procedure**

The following 2 groups are established in this study.

**(1) Rivaroxaban administration + physical therapy group (rivaroxaban group).**

A rivaroxaban is orally administered for about 3 months by the dosage regimen indicated by the package insert, and exhaustive guidance about physical therapy including ankle exercise and walking as well the use of graduated compression stockings (knee length、ankle pressure 15 to 30 mmHg) was given.

The initial administration is made as soon after allocation as possible. A rivaroxaban 15mg is administered orally and twice a day for the first 21 days (initial treatment period). Thereafter, the dose is reduced to 15 mg once a day, and the administration is continued for 69±14 days (maintenance period). The administration is performed a total of 90±14 days with the initial treatment and maintenance periods combined.

**(2) Physical therapy group.**

Exhaustive guidance about physical therapy including leg elevation, massage, ankle exercise, and walking the use of graduated compression stockings as well is given.

Graduated compression stockings are used for 90±14 days in both the rivaroxaban and physical therapy groups. If the use of graduated compression stockings or the continuation is unavoidably difficult, they may be replaced with elastic bandages or be discontinued. Concerning the state of their use, the number of days of their use and mean duration of their use per day are recorded in the case report form on each visit after the beginning of the study.

#### Procedure for reducing the dose of rivaroxaban.

In the initial treatment period, the dose may be reduced to the maintenance dose at the discretion of the investigator if there is concern over the risk of bleeding such as low body weight, kidney dysfunction, and the concomitant use of antithrombotic drugs or non-steroidal anti-inflammatory drugs (NSAIDs).

#### Procedure for suspension of rivaroxaban administration.

The administration of rivaroxaban may be suspended in the event of invasive treatment including surgery or if there is medical necessity (the occurrence of bleeding events, unavoidable use of drugs the concomitant use of which is prohibited, etc.). In the treatment for the adverse events, appropriate measures based on the guidelines and general clinical practice are taken. After the resolution of the events including bleeding, the judgment of whether the administration should be resumed or discontinued is made by the investigator. The period of cessation should be minimized.

## **Supplementary Appendix 6: Endpoints and Definition of the Endpoints**

### **Primary endpoint**

The occurrence of any of symptomatic or asymptomatic proximal DVT, symptomatic PE, and fatal PE within Day 90 (± 14 days) after the beginning of the study (combined evaluation item).

### **Secondary endpoint**

- recurrent isolated distal DVT
- symptomatic proximal DVT
- asymptomatic proximal DVT
- symptomatic PE (non-fatal or fatal)
- major bleeding
- clinically relevant non-major bleeding

These endpoints are evaluated within Day 14 (±3), 90 (±14), or 365 (±28).

### **Definitions for endpoint**

The following definitions are applied as evaluation criteria to be used by the Clinical Events Committee for the identification of events suspected to be PE or DVT.

**PE that corresponds to any of the following:**

- (Novel) intravascular filling defect detected by CT scan in lung segments or more proximal areas
- (Novel) intravascular filling defect, widening of existing filling defect, or novel sudden vascular collapse 2.5 mm or greater detected by pulmonary arteriography
- At least 75% (novel) filling defect of the pulmonary blood flow detected by lung scintigraphy in areas showing (a high probability of) normal ventilation image

**DVT that corresponds to any of the following:**

- Abnormal findings on whole-leg US (including longitudinal extension of thrombus) in areas with normal whole-leg US findings at registration, or, if the whole-leg US findings at registration were abnormal (not completely compressible), increase in the thrombus diameter by complete compression (an increase of 4 mm or more compared with a previous measurement in the proximal type; an increase of 50% or more in the calf-localized type, by complete compression)
- Enlargement of intravascular filling defect detected by venography or CT scan, novel intravascular filling defect, or dilation of veins in invisible areas accompanied by sudden vascular occlusion

**Fatal PE**

- PE diagnosed by an objective diagnostic method or autopsy

**Probable DVT, PE, or fatal PE**

- Even when an objective diagnosis has not been made, if DVT or PE is suspected, and anticoagulant therapy is introduced at a therapeutic dose for its treatment, the condition is defined as probable DVT or PE.
- Death in which PE cannot be excluded as its cause without demonstration of other causes

**Major bleeding**

Major bleeding is defined as a bleeding complication met any of the following criteria:

- A decrease in hemoglobin of ≥2 g/dL
- Necessity of transfusion of 4 units (equivalent to 800 mL, corresponds to 2 units overseas) or more of packed red cells or whole blood.
- Bleeding at important organs: intracranial hemorrhage, intramedullary hemorrhage, intraocular hemorrhage, pericardial bleeding
- Intraarticular bleeding, intramuscular bleeding accompanied by compartment syndrome, retroperitoneal bleeding
- Bleeding leading to death

**Clinically relevant non-major bleeding**

Clinically relevant non- major bleeding does not meet the criteria of major bleeding but is clinically relevant. It includes those requiring medical intervention, unscheduled visits, telephone inquiries, or discontinuation (suspension) of rivaroxaban administration, those accompanying unpleasant symptoms such as pain, and those interfering with daily living.

Examples of the bleeding events are as follows:

- Bleeding from the nose that persists for 5 minutes or longer, is recurrent (bleeding occurring 2 or more times within 24 hours; evens that leave only spots of blood on handkerchief are excluded), or requires some intervention (packing, electric coagulation, etc.)
- Gingival bleeding occurring spontaneously (unrelated to tooth brushing or eating) or persisting for 5 minutes or longer
- Gross hematuria occurring spontaneously or persisting for more than 24 hours after catheterization or surgery and bleeding from the urogenital tract
- Gross gastrointestinal bleeding accompanied by at least 1 episode of clinically evident of melena/hematemesis
- Rectal bleeding (at least a few spots of blood are observed on toilet paper)
- Hemoptysis (at least a few spots are observed in sputum)
- Intramuscular hematoma
- Subcutaneous hematoma with a size exceeding 25 cm^2^ or, if there is an inducing factor such as trauma, exceeding 100 cm^2^.
- Bleeding with multiple sources

# **Supplementary Tables**

## **Table S1: Consort 2010 checklist compliance for our randomized trial**

| **Section/Topic** | **Item No** | **Checklist item** | **Reported on page No** |
| --- | --- | --- | --- |
| **Title and abstract** | | | |
|  | 1a | Identification as a randomised trial in the title | 1 |
|  | 1b | Structured summary of trial design, methods, results, and conclusions (for specific guidance see CONSORT for abstracts) | 4-5 |
| **Introduction** | | | |
| Background and objectives | 2a | Scientific background and explanation of rationale | 6 |
|  | 2b | Specific objectives or hypotheses | 6 |
| **Methods** | | | |
| Trial design | 3a | Description of trial design (such as parallel, factorial) including allocation ratio | 6-8 |
|  | 3b | Important changes to methods after trial commencement (such as eligibility criteria), with reasons | Not applicable |
| Participants | 4a | Eligibility criteria for participants | 7-8 |
|  | 4b | Settings and locations where the data were collected | 6 |
| Interventions | 5 | The interventions for each group with sufficient details to allow replication, including how and when they were actually administered | 8 |
| Outcomes | 6a | Completely defined pre-specified primary and secondary outcome measures, including how and when they were assessed | 9-10 |
|  | 6b | Any changes to trial outcomes after the trial commenced, with reasons | Not applicable |
| Sample size | 7a | How sample size was determined | 10 |
|  | 7b | When applicable, explanation of any interim analyses and stopping guidelines | Not applicable |
| Randomisation: |  |  |  |
| Sequence generation | 8a | Method used to generate the random allocation sequence | 8 |
|  | 8b | Type of randomisation; details of any restriction (such as blocking and block size) | 8 |
| Allocation concealment mechanism | 9 | Mechanism used to implement the random allocation sequence (such as sequentially numbered containers), describing any steps taken to conceal the sequence until interventions were assigned | 8 |
| Implementation | 10 | Who generated the random allocation sequence, who enrolled participants, and who assigned participants to interventions | 8 |
| Blinding | 11a | If done, who was blinded after assignment to interventions (for example, participants, care providers, those assessing outcomes) and how | 7  An independent committee, blinded to treatment assignments, adjudicated all prespecified events. |
|  | 11b | If relevant, description of the similarity of interventions | Not applicable |
| Statistical methods | 12a | Statistical methods used to compare groups for primary and secondary outcomes | 10 |
|  | 12b | Methods for additional analyses, such as subgroup analyses and adjusted analyses | 10 |
| **Results** | | | |
| Participant flow (a diagram is strongly recommended) | 13a | For each group, the numbers of participants who were randomly assigned, received intended treatment, and were analysed for the primary outcome | 10-11 |
|  | 13b | For each group, losses and exclusions after randomisation, together with reasons | 11 |
| Recruitment | 14a | Dates defining the periods of recruitment and follow-up | 10 |
|  | 14b | Why the trial ended or was stopped | 10 |
| Baseline data | 15 | A table showing baseline demographic and clinical characteristics for each group | Table1 |
| Numbers analysed | 16 | For each group, number of participants (denominator) included in each analysis and whether the analysis was by original assigned groups | Figure1 |
| Outcomes and estimation | 17a | For each primary and secondary outcome, results for each group, and the estimated effect size and its precision (such as 95% confidence interval) | 11-12, Table2 |
|  | 17b | For binary outcomes, presentation of both absolute and relative effect sizes is recommended | Not applicable |
| Ancillary analyses | 18 | Results of any other analyses performed, including subgroup analyses and adjusted analyses, distinguishing pre-specified from exploratory | Not applicable |
| Harms | 19 | All important harms or unintended effects in each group (for specific guidance see CONSORT for harms) | 11-12, Table2-3 |
| **Discussion** | | | |
| Limitations | 20 | Trial limitations, addressing sources of potential bias, imprecision, and, if relevant, multiplicity of analyses | 13-14 |
| Generalisability | 21 | Generalisability (external validity, applicability) of the trial findings | 13-14 |
| Interpretation | 22 | Interpretation consistent with results, balancing benefits and harms, and considering other relevant evidence | 12-14 |
| **Other information** | | |  |
| Registration | 23 | Registration number and name of trial registry | 2 |
| Protocol | 24 | Where the full trial protocol can be accessed, if available | The full trial protocol is available upon request. Interested parties are encouraged to contact our facility via email for access. |
| Funding | 25 | Sources of funding and other support (such as supply of drugs), role of funders | 2 |

## **Table S2. Reasons for performing ultrasound**

| **Reasons for performing ultrasound, No. (%)** | **Total**  **(N=87) ^a^** |
| --- | --- |
| Non-operative screening for patients at risk of VTE | 38 (43.7) |
| Pre-operative screening for patients at risk of VTE ^b^ | 21 (24.1) |
| Post-operative screening for patients at risk of VTE | 12 (13.8) |
| Suspected DVT based on the symptoms | 16 (18.4) ^c^ |

^a^ Reasons for performing ultrasound are described in the intention-to-treat population.

^b^ High risk of VTE included active cancer, bedridden status, or hospital admission.

^c^ Although ultrasound was performed for 16 patients suspicious of DVT based on the symptoms, as a result of the test, symptoms in seven patients turned out to be due to another reason unrelated to IDDVT.

VTE, venous thromboembolism; DVT, deep vein thrombosis, IDDVT, isolated distal deep vein thrombosis.

## **Table S3. Reasons for selecting the initial lower dosing of rivaroxaban**

| **Reasons, No. (%)** | **Total**  **(N=42)** |
| --- | --- |
| Bleeding risk considered high * | 41 (97.6) |
| VTE recurrence risk considered not particularly high | 1 (2.4) |

* High bleeding risk includes factors such as active cancer, advanced age, anemia, previous bleeding history, concurrent use of medications increasing bleeding risk, etc.

VTE, venous thromboembolism.

## **Table S4. Efficacy and safety clinical outcomes at Day 14 (± 3 days)**

|  | **Rivaroxaban**  **(N=42)** | | **Physical therapy**  **(N=45)** | | **Absolute risk reduction** |
| --- | --- | --- | --- | --- | --- |
|  | **No. (%)** | **95% CI ^a^** | **No. (%)** | **95% CI ^a^** | **% (95% CI) ^b^** |
| **Thrombotic events** |  |  |  |  |  |
| Composite outcome of symptomatic or asymptomatic proximal DVT or symptomatic PE | 0 (0) | 0 – 8.4 | 0 (0) | 0 – 7.9 | 0 |
| Symptomatic PE and proximal DVT | 0 (0) | 0 – 8.4 | 0 (0) | 0 – 7.9 | 0 |
| Symptomatic PE | 0 (0) | 0 – 8.4 | 0 (0) | 0 – 7.9 | 0 |
| Symptomatic proximal DVT | 0 (0) | 0 – 8.4 | 0 (0) | 0 – 7.9 | 0 |
| Asymptomatic proximal DVT | 0 (0) | 0 – 8.4 | 0 (0) | 0 – 7.9 | 0 |
| Symptomatic or asymptomatic IDDVT | 1 (2.4) | 0.1 – 12.6 | 8 (17.8) | 8.0 – 32.1 | 15.4 (2.5 – 29.8) |
| **Bleeding events** |  |  |  |  |  |
| Major bleeding | 1 (2.4) | 0.1 – 12.6 | 0 (0) | 0 – 7.9 | -2.4 (-12.6 – -5.8) |
| Clinically relevant bleeding | 3 (7.1) | 1.5 – 19.5 | 0 (0) | 0 – 7.9 | -7.1 (-19.5 – 1.3) |
| **All-cause death** | 0 (0) | 0– 8.4 | 0 (0) | 0 – 7.9 | 0 |

Categorical variables are presented as numbers and percentages. Regarding secondary endpoints, the rate of the occurrence of events is reported as the number of patients developing at least one event divided by the total number of patients in the intention-to-treat analysis set. Absolute Risk Reduction is calculated as the event rate in the physical therapy group minus the event rate in the rivaroxaban group. Clinically relevant bleeding comprises major bleeding and clinically relevant non-major bleeding.

^a^ The 95% CI of the rate of occurrence was calculated using the Clopper-Pearson method.

^b^ The 95% CI for the risk difference is based on the score statistic.

CI, confidence interval; DVT, deep vein thrombosis; PE, pulmonary embolism; IDDVT, isolated distal deep vein thrombosis.

## **Table S5. Efficacy and safety clinical outcomes at Day 365 (± 28 days)**

|  | **Rivaroxaban**  **(N=42)** | | **Physical therapy**  **(N=45)** | | **Absolute risk reduction** |
| --- | --- | --- | --- | --- | --- |
|  | **No. (%)** | **95% CI ^a^** | **No. (%)** | **95% CI ^a^** | **% (95% CI) ^b^** |
| **Thrombotic events** |  |  |  |  |  |
| Composite outcome of symptomatic or asymptomatic proximal DVT or symptomatic PE ^c^ | 1 (2.7) | 0.1 – 14.2 | 2 (4.9) | 0.6 – 16.5 | 2.2 (-9.8 – 14.2) |
| Symptomatic PE and proximal DVT ^c^ | 1 (2.7) | 0.1 – 14.2 | 2 (4.9) | 0.6 – 16.5 | 2.2 (-9.8 – 14.2) |
| Symptomatic PE ^c^ | 0 (0) | 0 – 8.4 | 0 (0) | 0 – 7.9 | 0 |
| Symptomatic proximal DVT ^c^ | 1 (2.7) | 0 – 8.4 | 2 (4.9) | 0.6 – 16.5 | 2.2 (-9.8 – 14.2) |
| Asymptomatic proximal DVT ^c^ | 0 (0) | 0 – 8.4 | 0 (0) | 0 – 7.9 | 0 |
| Symptomatic or asymptomatic IDDVT ^d^ | 24 (63.2) | 46.0 – 78.2 | 16 (39.0) | 24.2 – 55.5 | -24.1 (-44.9 – -1.5) |
| **Bleeding events ^e^** |  |  |  |  |  |
| Major bleeding | 4 (10.3) | 2.9 – 24.2 | 1 (2.4) | 0.1 – 12.9 | -7.8 (-22.1 – 3.9) |
| Clinically relevant bleeding | 9 (23.1.) | 11.1 – 39.3 | 1 (2.4) | 0.1 – 12.9 | -20.6 (-37.1 – -6.1) |
| **All-cause death** | 6 (14.3) | 5.4 – 28.5 | 2 (4.4) | 0.5 – 15.1 | -9.8 (-24.6 – 3.1) |

Categorical variables are presented as numbers and percentages. Regarding secondary endpoints, the rate of the occurrence of events is reported as the number of patients developing at least one event divided by the total number of patients in the intention-to-treat analysis set. Absolute Risk Reduction is calculated as the event rate in the physical therapy group minus the event rate in the rivaroxaban group. Clinically relevant bleeding comprises major bleeding and clinically relevant non-major bleeding.

^a^ The 95% CI of the rate of occurrence was calculated using the Clopper-Pearson method.

^b^ The 95% CI for the risk difference is based on the score statistic.

^c^ Data missing for 5 patients in the rivaroxaban group and 4 patients in the physical therapy group.

^d^ Data missing for 4 patients in the rivaroxaban group and 4 patients in the physical therapy group.

^e^ Data missing for 3 patients in the rivaroxaban group and 4 patients in the physical therapy group.

CI, confidence interval; DVT, deep vein thrombosis; PE, pulmonary embolism; IDDVT, isolated distal deep vein thrombosis.
